# Efficacy and Tolerability of Transoral Sialolithotomy in Non‐Palpable Submandibular Lithiasis

**DOI:** 10.1002/lary.70027

**Published:** 2025-08-06

**Authors:** Marine Bourtoul, Hugo Frandjian, Jean‐Marc Foletti, Adèle Brotons, Quentin Hennocq, Cyrille Chossegros, Nicolas Graillon

**Affiliations:** ^1^ APHM, Conception University Hospital Department of Oral and Maxillofacial Surgery Marseille France; ^2^ Aix Marseille Université Marseille France; ^3^ Université Gustave Eiffel Marseille France; ^4^ Laboratoire de biomécanique appliquée (LBA) Marseille France; ^5^ Centre National de Recherche Scientifique (CNRS) Marseille France; ^6^ Etablissement français du sang (EFS) Marseille France; ^7^ Anthropologie bio‐culturelle, Droit, Ethique et Santé (ADES) Marseille France; ^8^ Sorbonne Université Paris France; ^9^ Department of Maxillo‐Facial Surgery Hôpital Pitié‐Salpêtrière, Assistance Publique des Hôpitaux de Paris Paris France

**Keywords:** minimally‐invasive surgery, palpation reliability, sialolithiasis submandibular, sialolithotomy

## Abstract

**Objective:**

Transoral sialolithotomy (TSL) is the oldest technique used to remove submandibular stones. While it was initially limited to palpable and anterior stones, its indications have broadened. Although non‐palpability has traditionally been considered a contraindication, our experience suggests TSL remains feasible in such cases. This study aimed to evaluate the efficacy and safety of TSL for non‐palpable submandibular lithiasis. Secondary objectives were to identify distinguishing characteristics of non‐palpable stones and factors associated with surgical success.

**Methods:**

We conducted a retrospective, monocentric study at Conception University Hospital, Marseille, including all patients treated with TSL between April 2014 and January 2024. Demographic and clinical data were collected: stone laterality, size, location, palpability, multiplicity, operative time, and complications. Univariate and multivariate logistic regression analyses were used to assess factors influencing success.

**Results:**

A total of 457 patients were included (380 with palpable stones, 77 with non‐palpable). TSL achieved an 87% success rate for non‐palpable stones, with a complication rate under 1%. Non‐palpable stones were significantly smaller (mean 5.3 mm vs. 9 mm). Palpability was strongly associated with success (OR = 11.03 [2.95–55.00], *p* < 0.001).

**Conclusion:**

TSL is an effective and safe option for non‐palpable submandibular stones. The absence of palpability alone should not exclude patients from this technique, even in the absence of sialendoscopy. However, patients should be informed of the increased risk of failure. Based on our surgical experience, systematic 3‐D imaging, general anesthesia for deep gland exposure, and pressurized saline flushing improve the success of TSL in non‐palpable stones.

**Level of Evidence:**

4.

## Graphical abstract

Transoral sialolithotomy has long been considered inappropriate for non‐palpable submandibular stones. In this retrospective cohort of 457 patients, we compared outcomes between palpable and non‐palpable stones, showing that the technique allows successful removal in 87% of non‐palpable cases, with no associated complications. This suggests it can be considered even in centers without access to endoscopic or fragmentation techniques.

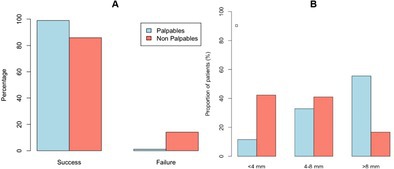

## 1 Introduction

Sialolithiasis is the most common disorder affecting the major salivary glands, with the submandibular gland being most frequently affected (approximately 80%) [1]. Histological [2] and scintigraphic [3] studies have shown that the gland recovers structurally and functionally after stone removal, paving the way for organ‐preserving techniques.

Since the 1990s, minimally invasive options are now first‐line therapy. Transoral sialolithotomy (TSL) preserves the gland and achieves high success, although the technique has traditionally been avoided when no stone is palpable [4, 5]. Interventional sialendoscopy is incision‐free and ideal for small, mobile stones, but requires dedicated equipment, a steep learning curve, and carries a measurable risk of duct injury [6, 7]. Laser lithotripsy extends endoscopic management to larger, immobile stones, yet it lengthens anesthesia and adds a specific hazard of thermal damage to the duct [6, 7, 8]. Extracorporeal shock‐wave lithotripsy is fully non‐invasive but usually requires several sessions and is not widely available [9]. Finally, the combined endoscopic‐transoral approach is often recommended for deep hilar or intraglandular calculi; it delivers very high clearance with minimal nerve morbidity, but at the price of longer operative time and dual instrumentation [10, 11, 12].

Among all extraction techniques, TSL is the oldest and most widely accessible. Initially reserved for easily accessible stones, it can now be used for posterior stones [13]. Although stone palpability was once considered essential [14, 15, 16, 17, 18], our experience shows TSL remains feasible even in cases of non‐palpable stones; that this criterion alone should not limit its indication.

Therefore, the primary objective of this study was to evaluate the efficacy and tolerability of TSL for non‐palpable submandibular lithiasis. The secondary objectives included assessing the characteristics differentiating a non‐palpable stone from a palpable stone and identifying the factors that were associated with surgical success.

## 2 Materials and Methods

We conducted a monocentric retrospective study that included all patients who underwent TSL at the Conception University Hospital in Marseille, France, between April 2014 and January 2024. The procedures were performed by three experienced surgeons, using the technique previously described by Carbonnel et al. [19]. Patients who underwent emergency surgery for acute infections were excluded (*n* = 11). Data collection included demographic (e.g., gender, age) and stone‐related variables, such as stone laterality, diameter, location, and palpability during the consultation. Stone diameter and location were assessed on preoperative computed tomography (CT) or cone‐beam computed tomography (CBCT). A posterior stone was defined as a hilar or intraglandular stone, as previously explained by Foletti et al. [20]. The presence of multiple stones and the operative time were also recorded. A failed procedure was defined as a non‐removal stone responsible for the symptoms. In cases of multiple stones, even if one or more were removed, the procedure was still considered a failure if the symptoms persisted. The tolerability of the procedure was assessed by the operative time and the postoperative complications at 1 month, 3 months, and 1 year. Only complications requiring reoperation or persisting after 1 year were considered. Anterior stones were usually treated under local anesthesia, while general anesthesia was preferred for posterior stones. This was a retrospective study of pre‐existing anonymized data and was exempt from ethics committee approval. Written informed consent was obtained from the patients.

## 3 Statistical Analysis

Statistical analyses were performed using the RStudio software (version 2024.04.2+764). A 5% alpha risk error was used. Data normality was assessed (Shapiro–Wilk test). For variables following a normal distribution, parametric mean comparison tests were used, such as the Student's *t*‐test for two groups and analysis of variance (ANOVA) for means comparisons between more than two groups. When normality assumptions were not met, non‐parametric mean comparison tests, such as the Wilcoxon–Mann–Whitney U test, were applied. Proportions and distributions were evaluated using Chi‐square (*χ* ^2^) tests. Finally, we performed univariate and multivariate logistic regression to identify variables with a significant influence on the success rate of stone removal. Variables with a significant *p*‐value in the univariate analysis were included in the multivariate analysis as potential confounding factors.

## 4 Results

We included 457 patients (228 men and 229 women; mean age 48.5 ± 16 years). The average stone size was 8.3 ± 4.7 mm. The majority of the stones were located in the posterior third (65.6%). Of the 457 patients, 380 had clinically palpable stones, and 77 had non‐palpable stones at preoperative consultation.

### 4.1 Efficacy (Table 1 and Figure 1A)

**TABLE 1:** Patient and stone characteristics.

	Total *N* = 457	P *n* = 380	NP *N* = 77	*p*
Male	228 (49.9%)	194 (51.1%)	34 (44.2%)	NS
Female	229 (50.1%)	186 (48.9%)	43 (55.8%)	^a^
Age (Mean ± SD)	48.57 ± 16.17	49.43 ± 15.41	44.34 ± 19.03	0.0148^a^
Interval	[12.26; 90.96]	[12.26; 90.96]	[12.68; 80.81]	
Laterality: right	239 (52.3%)	200 (52.6%)	39 (50.6%)	NS
Diameter (Mean ± SD)	8.34 ± 4.74	8.96 ± 4.88	5.29 ± 2.28	< 0.0001^a^
Interval	[1; 38.8]	[1; 38.8]	[2; 13]	< 0.0001^b^
< 4 mm	77 (16.8%)	44 (11.6%)	33 (42.9%)	
4–8 mm	157 (34.3%)	125 (32.9%)	32 (41.6%)	
> 8 mm	223 (48.9%)	211 (55.5%)	12 (15.6%)	
Location				NS
Anterior third	75 (16.4%)	69 (18.2%)	6 (7.8%)	
Middle third	82 (17.9%)	68 (17.9%)	14 (18.2%)	
Posterior third	300 (65.6%)	243 (63.9%)	57 (74%)	
Multiple stones (≥ 2)	131 (28.7%)	118 (31.1%)	13 (16.8%)	0.0121^a^
Operative time (Mean ± SD)	34.43 ± 17.05	33.43 ± 16.65	39.01 ± 18.18	< 0.0001^a^
Interval	[1; 120]	[1; 120]	[13; 96]	
Failure	14 (3.1%)	4 (1.1%)	10 (13%)	< 0.0001^b^ ^+^
Success	443 (96.9%)	376 (98.9%)	67 (87%)	

*Note*: Age is expressed in years, diameter in millimeters, and operative time in minutes.

Abbreviations: NP, non‐palpable; NS, not significant; P, palpable; SD, standard deviation.

^a^ Wilcoxon signed‐rank test.

^b^ Chi‐square test (+: with Yates correction).

**FIGURE 1:**
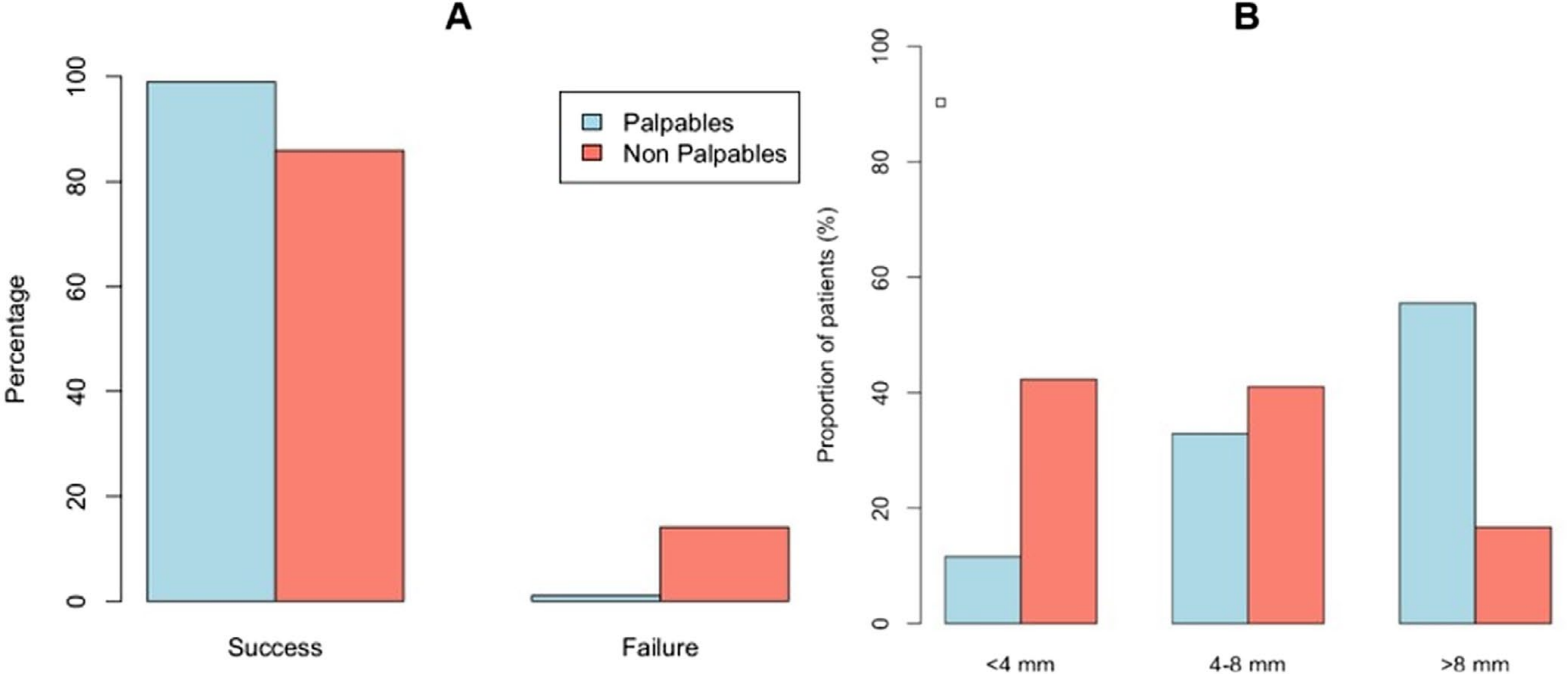
(A) Distribution of palpable and non‐palpable stones among successful and failed procedures. (B) Distribution of palpable and non‐palpable stones across different stone size categories (< 4 mm, 4–8 mm, and > 8 mm). [Color figure can be viewed in the online issue, which is available at www.laryngoscope.com]

A total of 14 failures occurred (3.1%): 10 (13%) in the non‐palpable stone group and 4 (1.1%) in the palpable stone group. The overall success rate was 96.9%. Considering the non‐palpable and palpable stones, the success rate was 87% and 98.9%, respectively (*p* < 0.05).

### 4.2 Tolerability

The mean operative time was longer for non‐palpable stones (39 min) than palpable ones (33 min) (*p* = 0.02) (Table 1). Three patients (< 1%) experienced postoperative complications. In the palpable stone group, one patient experienced lingual hypoesthesia, which was resolutive after 18 months. In the non‐palpable stone group, one patient developed a floor‐of‐mouth abscess requiring surgical drainage, while another had a persistent ranula.

### 4.3 Non‐Palpable Stone Characteristics (Table 1)

Non‐palpable stones were smaller (with average diameter = 5.3 mm), relative to the palpable stones (average diameter = 9 mm; *p* < 0.05). Of note, 16% of the non‐palpable stones measured > 8 mm, compared to 56% of the palpable stones (Figure 1B). In both groups, the posterior third was the preferential location. The proportion of stones in this region was higher in the non‐palpable group (74%) than in the palpable group (64%). In contrast, anterior stones represented 18% of palpable stones and 8% of non‐palpable stones, with no statistically significant difference.

### 4.4 Factors Associated With Surgical Success (Table 2)

**TABLE 2:** Logistic regression results for the success of stone removal.

	Univariate			Multivariate		
	OR	95% CI	*p*	OR	95% CI	*p*
Gender: females	1.520	[0.539–4.603]	0.434			
Age (years)	0.992	[0.960–1.025]	0.638			
Operative time (min)	0.961	[0.938–0.986]	**0.001**	0.963	[0.937–0.990]	**0.006**
Side: right	1.664	[0.590–5.038]	0.342			
Palpability	15.43	[5.112–56.99]	**< 0.001**	11.03	[2.947–55.00]	**< 0.001**
Multiple stones	2.641	[0.716–17.05]	0.205			
Location						
Median	0.999	[0.303–4.494]	0.999			
Anterior	2.807	[0.533–51.72]	0.327			
Diameter (mm)	1.294	[1.082–1.602]	**0.010**	1.172	[0.944–1.522]	0.199

*Note*: The bold values indicate statistically significant *p* < 0.05.

In the multivariate model, the palpability of the stone was the strongest predictor of success, with an odds ratio (OR) of 11.03 (95% CI [2.95–55.00], *p* < 0.001). Similarly, shorter operative time was associated with higher success (OR = 0.963; 95% CI [0.937–0.990], *p* = 0.006). Although stone diameter was significant in univariate analysis (OR = 1.294; 95% CI [1.082–1.602]; *p* = 0.010), it was no longer significant in the multivariate model (*p* = 0.199). The location showed no significant association with surgical success. However, 71.4% of the stones that could not be removed were located in the posterior third, 28.6% in the middle third, and none were in the anterior third. The presence of multiple stones was not associated with an increased risk of failure, accounting for 21.4% of failures (3/14) and 29.1% of successes (129/443).

### 4.5 Follow‐Up and Causes of Failure According to Palpability

**FIGURE 2:**
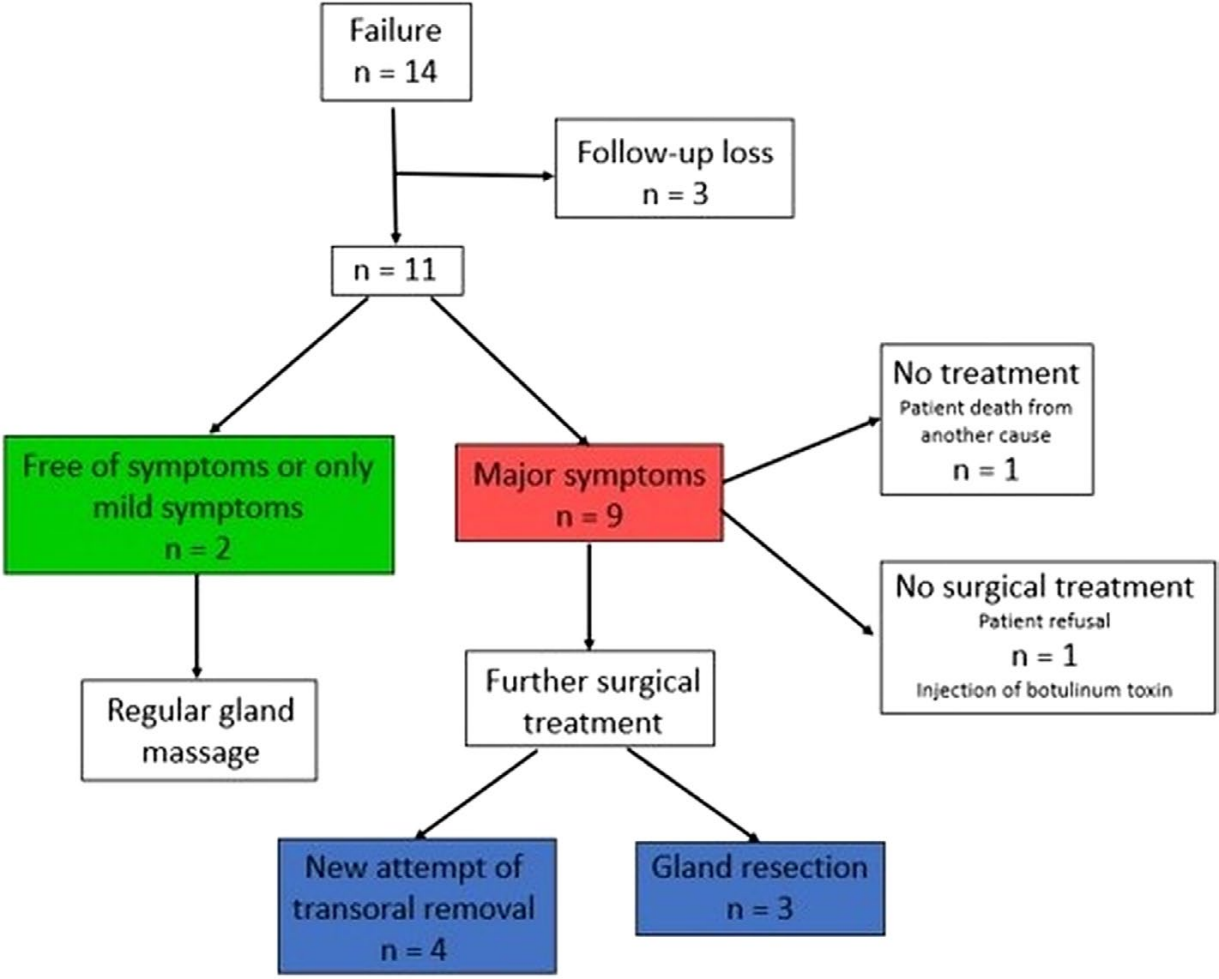
Follow‐up of failed procedures. [Color figure can be viewed in the online issue, which is available at www.laryngoscope.com]

The median follow‐up duration was 1.3 months. Fifty nine percentage of patients were seen at 1 month, 37% at 3 months, and 21% at 12 months. Notably, 27% of patients had no documented follow‐up. Figure 2 illustrates the follow‐up of patients who experienced failed stone removal. In our study, 21.4% (3/14) of failed TSL cases ultimately required gland excision.

Regarding the causes of failures according to palpability: in NP stones, the predominant cause was “stone not found” (8/11), half of which were associated with severe floor‐of‐the‐mouth fibrosis. Deep intraglandular impaction accounted for two cases, and a single failure resulted from a residual symptomatic stone/fragment. In contrast, failures in the P group were related to residual symptomatic stone/fragments in two of the three cases, whereas the remaining case was cause by a deep impaction.

## 5 Discussion

Our study confirmed that TSL is an effective stone removal technique, with an overall success rate of 97%, and 99% for palpable stones. The complication rate was very low (< 1%). These findings are consistent with those reported in the literature [18, 21, 22, 23, 24, 25], where success rates frequently approach or exceed 90%. Our study also demonstrates that this technique is suitable for non‐palpable stones, with a success rate of 87%. However, the management of non‐palpable stones by TSL has only recently begun to attract attention and remains poorly documented in the literature. Two recent studies reported success rates of 80% [26] and 94% [10]; but both focused solely on posterior stones and involved small cohorts (32 and 17 patients, respectively). To our knowledge, this is the first study to analyze the characteristics of non‐palpable stones and to include such a large sample (*n* = 77).

In our study, two variables were significantly associated with surgical success in the multivariate analysis: stone palpability and operative time. Palpability was the strongest predictor, with an odds ratio of 11.03 (95% CI [2.95–55.00], *p* < 0.001), consistent with the findings reported by Park et al. [18]. Operative time was also associated with success but should be interpreted as a confounding factor, reflecting technically demanding cases more prone to failure. Stone diameter was not statistically significant in the multivariate analysis but was significant in the univariate model. This suggests that its potential impact might be mediated by other factors, such as palpability. A larger sample could help clarify whether this variable independently influences surgical success. Previous observational studies have also explored associations between stone characteristics and surgical outcomes, although without adjusting for potential confounders. For instance, Zenk et al. [22] reported a 100% success rate for stones located in the duct; whereas, for those located in the hilum or parenchyma, the success rates were 91% for single stones and 64% for multiple stones. Shashinder et al. [5] similarly observed a higher likelihood of submandibulectomy in patients with multiple stones or with stones located in the posterior third of the duct. In our study, neither the location nor the presence of multiple stones was identified as independent factors associated with failed surgery. Nevertheless, it is interesting to note that among the failures, more than seven of 10 stones were located in the posterior third. In contrast, all stones located in the anterior third (*n* = 75) were successfully removed. Similarly, of the 14 failures recorded, three involved patients with multiple stones, whose failure was attributed to the persistence of symptoms; a finding that highlights the fact that the presence of multiple stones remains a technical challenge. In such cases, sialendoscopy may be useful for visualizing the entire salivary duct system and detecting residual fragments. However, in our experience, the standardized retrograde pressurized flushing protocol we use is generally effective in minimizing this risk.

Stone palpability was long viewed as the main determinant when choosing treatment, discouraging TSL because of presumed failure risk. Our data show that this subjective, examiner‐dependent parameter should not, by itself, preclude TSL. During consultation, palpation can be challenging due to gag reflexes, pain, or fibrosis/scarring mimicking a stone. Our study revealed that the smaller the stones, the higher the risk of not being detected during clinical examination. This is logically expected, as their small size can easily be concealed by the thickness of the overlying tissues. Intraoperatively, after soft tissue dissection, tactile and visual recognition becomes easier, facilitating stone detection. In our study, stones located in the posterior region appeared more difficult to detect, as their proportion was higher in the non‐palpable group. Although not statistically significant in this study, a larger patient sample may confirm this trend. Under general anesthesia, detection is easier because the gland can be elevated and the floor musculature is relaxed. Thus, preoperative non‐palpability does not necessarily indicate intraoperative non‐palpability. This same reasoning seems to have guided the therapeutic strategy reported by Ruiz et al. [21], who recommended verifying the palpability of stones > 5 mm under general anesthesia before considering a combined approach.

When the stone is not palpable preoperatively, the surgical technique must be adapted. The submandibular duct is first located approximately 1 cm behind the ostium, and dissection is then carried out toward the hilum and the gland, taking care to identify and protect the lingual nerve. General anesthesia with muscle relaxation and gentle gland elevation enhance intra‐operative palpation, so a stone impalpable in clinic can often be detected at this stage. If the stone remains impalpable, this usually indicates a deeply embedded and/or small intraglandular stone; an incision is then made directly at the hilum, and a pressurized saline flush is delivered to expel the calculus. Ultimately, only certain stones that are deeply embedded within the submandibular gland remain inaccessible to TSL.

However, knowing the exact location of the stone can enhance the probability of success during surgical extraction. In our study, all patients underwent 3D imaging. In addition to their excellent diagnostic performance [27, 28], these techniques allow highly accurate localization of stones based on the surrounding anatomical structures. Lee et al. [10] proposed using the submylohyoid line, which is easily identifiable on coronal sections, as a landmark to anticipate extraction difficulty. Stones located below this line are more difficult to access, which may compromise their removal through either a transoral or sialendoscopic approach. In our study, although location was not identified as a risk factor for failure, it is worth noting that three of the failures involved stones deeply embedded within the gland.

In addition, some authors recommend performing imaging on the day of surgery to minimize the risk of migration or spontaneous expulsion of the stone(s) that may occur in the interim [29]. As a reminder, the stone was not found in eight patients. In four of these cases, no obvious explanation was identified; therefore, a spontaneous and unnoticed elimination of the stone prior to surgery remains a plausible hypothesis. This is supported by the fact that among the 14 failures, two patients reported few or no postoperative symptoms, and three were lost to follow‐up. For the latter, the lack of postoperative data—likely due to our institutional organization—may actually reflect a favorable outcome. Indeed, many of our patients come from distant areas and, in the absence of complications, typically continue their follow‐up with their referring physician, which limits the collection of postoperative data. In this context, and given that immediate preoperative imaging is not always feasible, it seems essential to systematically ask patients about any recent disappearance of symptoms prior to surgery in order to avoid unnecessary surgical exploration.

Several therapeutic alternatives to TSL are available for the management of salivary stones. Among them, interventional sialendoscopy enables the removal of certain stones under endoscopic guidance. Used alone with instruments such as forceps or retrieval baskets, sialendoscopy is best suited to mobile intraductal calculi < 5 mm, offering a gland‐preserving, minimally invasive option; however, it requires dedicated equipment, a steep learning curve, and carries a 2%–6% risk of complications such as duct perforation, fibrosis, or stenosis [6, 7, 11]. Its effectiveness is also limited in tortuous or fibrotic ducts, and its cost and maintenance requirements restrict widespread availability, particularly outside tertiary‐care centres [24, 30].

Moreover, its indications are actually limited, as it has been shown that fixed, large, irregularly shaped stones located in the hilar or intraglandular region are associated with a significantly higher risk of failure [31]. In such cases, the technique must be adapted and may require either stone fragmentation or an associated TSL.

Fragmentation emerged in the 1990s and is now commonly performed using a Holmium:YAG laser [6]. Reported success averages 87% [6] while preserving the gland and avoiding external scars. Nevertheless, the method increases cost, carries a 2%–6% risk of duct perforation or thermal stenosis [6, 7, 8] and has certain limitations. Marchal et al. [7] demonstrated that increased operative time and the risk of requiring multiple procedures were correlated with stone size (for stones larger than 10 mm, the average operative time was 124 ± 44 min) and the presence of multiple stones. In contrast, in our study, the mean operative time for TSL was 35 min, which was consistent with previously published data [4, 5]. The procedure was 5 min longer for non‐palpable stones (39 min vs. 33 min), although this difference was not clinically relevant. Consequently, laser lithotripsy is best used as a stand‐alone option for visible yet immobile stones 5–8 mm [8, 20], and a recent study pinpointed 7.5 mm as the optimal upper threshold for single‐session clearance [8].

The combined approach, first described by Marchal [32] in 2007, uses sialendoscopic trans‐illumination to guide a precise mucosal incision. Two meta‐analyses report clearance rates of 93%–95% [11, 12]. A recent study by Lee et al. [10] specifically investigated non‐palpable posterior stones (hilar and intraglandular), comparing sialendoscopy with TSL. However, it is important to note that only 5% of patients were treated with sialendoscopy alone, meaning the analysis essentially compared the combined approach to TSL. The results showed comparable success rates between both techniques, with 98.6% for the combined approach and 94.1% for TSL alone, without a statistically significant difference. However, the combined approach was also associated with a longer operative time, casting doubt on its routine use. Additionally, although the authors reported a higher complication rate in the TSL group, these were all minor, transient effects (pain, edema, lingual numbness) with no functional impact and no need for revision surgery. Therefore, while the removal of non‐palpable stones by TSL may be technically more demanding—partly explaining its slightly lower success rate compared with palpable stones—its overall success remains high and, more importantly, comparable to other available therapeutic alternatives.

The main limitations of this study are its retrospective and monocentric design. Additionally, it would have been relevant to specify the proportion of stones that were non‐palpable during consultation but became palpable under general anesthesia. Among its strengths, this study included a large number of patients (*N* = 457) and an original topic. These findings open the door to future comparative studies. However, rather than focusing solely on success rates, such studies should instead aim to better define the specific clinical scenarios in which the addition of sialendoscopy to TSL provides a true clinical advantage, or in which sialendoscopy alone may be sufficient if available. Tailoring the approach to each patient's situation, rather than systematically resorting to a combined technique, could help optimize outcomes while ensuring a more pragmatic and resource‐conscious use of available tools.

In conclusion, TSL remains a reliable and effective technique for the removal of non‐palpable stones. Although the risk of failure is slightly higher due to increased technical complexity, it remains acceptable. Patients should be clearly informed of this risk prior to surgery. TSL remains a valuable option, particularly in centers without access to sialendoscopy, as it can be performed with standard surgical equipment.

## Ethics Statement

This study was a retrospective review of anonymized data and was exempted from ethics committee approval.

## Conflicts of Interest

The authors declare no conflicts of interest.

## Data Availability

The data that support the findings of this study are available from the corresponding author upon reasonable request.
